# How internet healthcare information overflow affects farmers’ healthcare consumption: insights from China

**DOI:** 10.3389/fpubh.2024.1380254

**Published:** 2024-04-22

**Authors:** Yu Ming, Chen Xia

**Affiliations:** ^1^Hunan Agricultural University, Changsha, China; ^2^Hunan Urban Professional College, Changsha, China

**Keywords:** farmers, internet, medical information spillover, healthcare consumption, mediating effects

## Abstract

**Introduction:**

In the context of the deep coupling and synergistic development of digital villages and healthy villages, the development of China’s rural society harbors a huge potential for medical and healthcare consumption.

**Methods:**

On the basis of theoretical research, a framework was constructed to analyze the influence mechanism of farmers’ medical and healthcare consumption in the context of Internet medical information overflow, and empirically examines the research and analysis framework by using the 2020 China Household Tracking Survey data with the OLS model, mediation effect model, and instrumental variable method.

**Results:**

It is found that Internet medical information spillover has a “crowding-in effect” on farmers’ healthcare consumption; Medical attendance behavior, economic capital utilize the intermediary effect between Internet medical information spillover and farmers’ healthcare consumption. And there is age group heterogeneity in the effect of Internet medical information spillover on farmers’ healthcare consumption, The ability of rural middle-aged and old-aged groups to recognize new things such as Internet medical information needs to be improved, so the overflow of Internet medical information will induce rural middle-aged and old-aged groups to generate a certain amount of medical and health care consumption. However, the impact on healthcare consumption is not sensitive to the youth cohort group.

**Discussion:**

The sinking of Internet medical resources should be accelerated in the future to promote the high-quality development of rural medical and health services, at the same time the “Internet + healthcare services” should be optimized to promote scientific and rational stratification of farmers’ access to healthcare, and economic capital for farmers’ access to health care should be improved in order to alleviate the burden of health care, etc.

## 1 Introduction

With the deepening of China’s healthcare system and the aging of the population, farmers’ healthcare consumption has continued to grow rapidly, but the structural imbalance in the allocation of healthcare resources has made the problem of “difficult and expensive access to healthcare” gradually emerge. There is a stark contrast in attendance and volume between primary and tertiary hospitals. Farmers recognize the “efficacy” of large hospitals, which lead to a “siphoning off” of health care choices for most rural households. While the additional costs of medical treatment brought about by “going out for medical treatment” will further aggravate the economic burden on farmers’ families. According to the “Statistical Bulletin on the Development of China’s Health and Wellness Program, 2022″, the total number of consultations and treatments at national healthcare institutions will reach 8.42 billion in 2022. However, the number of consultations at township health centers and community health service centers (stations) accounted for only 24.2 per cent of total consultations. And the nation’s total health costs grew from $458.6 billion in 2000 to $848.467 billion in 2022. The average hospital outpatient cost in 2022 is 342.7 yuan. 4.1% increase in current year prices over previous year, 3.0% increase in comparable prices (1). The mismatch of medical resources has led to a “spontaneous monopolistic” consumption of resources in large hospitals, where “the scarcity of goods is the most expensive,” which leads to “minor diseases in the community, major diseases into the hospital, rehabilitation back to the community” hierarchical diagnosis and treatment concept is difficult to penetrate into the hearts of grass-roots people. Against this background, it is worth pondering how to effectively guide rural residents to rationally seek medical treatment and control the unreasonable growth of rural residents’ health care consumption, so as to improve farmers’ literacy in resisting health risks.

Along with the gradual “introduction” of the “Internet+” medical and health service model into rural areas, the development of communication technology has “bred” the overflow of medical information on the Internet, and the “uncertainty” of medical information has also permeated the consumption of healthcare services in rural areas. The Outline of the “Healthy China 2030” Plan, issued by the Central Committee of the Communist Party of China and the State Council in 2016, points out that it focuses on rural and grassroots areas to promote the equalization of health and hygiene resources, building a health information service system and improving the health protection system to reduce the burden of individual contributions. The Opinions on the Implementation of the Rural Revitalization Strategy and the Strategic Plan for Rural Revitalization (2018–2022) issued by the CPC Central Committee and the State Council in 2018 point out that it is necessary to accelerate the construction of a healthy countryside, build the health cornerstone of rural revitalization in impoverished areas, accelerate the soundness of the relevant safeguard policy system, and build a long-term mechanism for stabilizing poverty eradication and preventing the return of poverty. The Twentieth Party Congress pointed out the need to build a strategic plan for a digital China, with the development of digital villages as a top priority, and the need to promote the construction of a healthy China, develop and strengthen the healthcare workforce, and focus efforts on rural areas and communities. National top-level design to guide digital technology resources to enhance farmers’ healthcare services in order to realize the deep coupling and synergistic development of “digital villages” and “healthy villages.”And under the dual requirements of digital healthy village construction and solidifying the results of poverty alleviation, reducing the burden of farmers’ medical and health care consumption is an important way to consolidate the results of poverty alleviation and to protect farmers’ healthy life (2). And China’s rural Internet infrastructure and digital applications are evolving, making Internet information overflow an important tool for rural residents’ healthcare consumption changes. According to data released by the China Internet Information Center, as of December 2022, the size of rural Internet users was 308 million; as of June 2022, rural internet penetration was 58.8%. At the same time, most older adult people can independently complete such online activities as presenting their health codes and travel cards, purchasing daily necessities, and searching for various types of information, and the technical conditions for the public to share the fruits of informationization are becoming more and more mature. It can be seen that the Internet information overflow has become an important engine to accelerate the healthcare and other consumption of rural residents (3).

Academic research on farmers’ healthcare consumption has centered on farmers’ incomes, government behavior, and the environment. First, from the perspective of farmers’ income, it is found that the difference in farmers’ income significantly affects healthcare consumption expenditures, and farmers with better household income status release healthcare demand and act on consumption expenditures (4, 5)^,^ “Unreasonable” medical prices and “non-transparent” charging systems can lead to farmers with little economic capital “not treating the sick” (6, 7). Secondly, from the perspective of government behavior it is found that government transfer revenues, the strength of public services, fiscal decentralization, and the medical insurance system all have a significant impact on farmers’ medical and health consumption expenditures (8, 9), Thirdly, from an environmental perspective it is found that, under the “Internet + healthcare” environment, Internet medical information provides farmers with online consultation interactions between doctors and patients, which meets the needs of farmers for “convenient access to medical care” and “convenient healthcare,” and enhances farmers’ health capital and human capital, thereby increasing farmers’ economic capital. However, the overflow of medical information from the Internet may increase the “uncertainty” of medical services, leading to the creation of medical induction effects, which in turn affects the relationship of trust between doctors and patients and the choice of medical care (10, 11)^.^ In turn, choices of medical care, etc., play a role in healthcare consumption. In the context of health care reform, the results of environmental management, such as rural sewage reform, toilet reform and domestic waste treatment, have had a significant impact on farmers’ health levels, prompting them to realize the importance of health, which in turn has changed the farmers’ medical and health consumption expenditures (12, 13). In addition, some scholars have also found that factors such as population age structure, health status, education level, gender, marital status, and potential demand for medical care have an influential relationship with farmers’ medical and health care consumption (14, 15). Although the existing literature is relatively rich, most of it mainly analyzes the role of economic income and choice of medical care in influencing farmers’ healthcare consumption. And big data, cloud computing, mobile Internet and other communication technologies have entered a period of rapid development, the patient’s disease diagnosis, disease treatment, disease prevention and other medical information through the Internet can be transmitted and diffused to the patient. However, previous literature has paid less attention to the impact of Internet healthcare information spillover and farmers’ healthcare consumption and its influencing mechanism, while further exploration of the endogeneity issues between Internet healthcare information spillover and farmers’ healthcare consumption as well as age heterogeneity is lacking.

The Internet healthcare information overflow referred to in this article refers to the process of transmitting and diffusing healthcare information to the population level through Internet healthcare information query, online healthcare information consultation, electronic medical records, electronic health records, telemedicine, etc. as online healthcare service modes, based on Internet and communication technologies. Consumer spending on health care in this paper mainly refers to residents’ spending on medical and health care medicines, medical-related devices, fitness and exercise, and service fees. Based on existing research, three questions are put forward. Firstly, does Internet medical information spillover affect farmers’ healthcare consumption? Secondly, does Internet healthcare information spillover act on farmers’ healthcare consumption by changing their healthcare behavior? Thirdly, does Internet healthcare information spillover work by altering farmers’ economic capital and thus their healthcare consumption? Therefore, based on the capital theory and information induced effect, both “medical behavior” and “economic capital” are incorporated into Internet medical information spillover and medical health consumption to construct an analytical framework for medical health consumption under the scenario of Internet medical information spillover. Using data from the latest edition of the China Family Tracking Survey (CFPS2020), a baseline regression model and a mediation model were adopted to empirically analyze the impact of Internet medical information spillover and farmers’ medical and healthcare consumption, as well as the mechanism of influence. Farmers’ healthcare consumption, structural allocation imbalance of healthcare services, and economic capital differentiation are creatively integrated into the Internet healthcare information spillover perspective, which can not only enrich the research literature on the social effects of rural areas under information technology, but also provide new policy tools and policy optimization paths to alleviate the pressure of farmers’ medical and health consumption, enhance their economic capital, and improve the hierarchical diagnosis and treatment system.

## 2 Materials and methods

### 2.1 Literature review and research hypothesis

#### 2.1.1 Research on the impact of internet medical information spillover on farmers’ healthcare consumption

Academic research on the impact of Internet medical information spillover on farmers’ healthcare consumption mainly centers on the “crowding-in effect” and “crowding-out effect.” From the perspective of crowding-in effect, Arrow introduced the uncertainty of consumption and asymmetry of information into the medical service field as early as 1963, and found that the market of medical service is very likely to have an “induced effect” on patients (16), while farmers have a weak sense of information prevention and serious information asymmetry between doctors and patients, they are highly susceptible to Internet information luring (17, 18). The ability of farmers to find optimal decisions is more dependent on their knowledge structure system and their ability to discriminate information. However, most farmers have a weak foundation in information technology, which makes it difficult to transform the “uncertainty” of Internet medical information into “certainty” (19). Doctors, on the other hand, have more professional knowledge and more say in the medical field, which can improve the certainty of Internet medical information. However, it is likely to exaggerate the patient’s condition due to its own economic interests, creating an “induced effect” on medical behavior, resulting in over-consumption of medical resources by the patient and increasing medical and healthcare consumption (20). From the perspective of the crowding-out effect, Internet information can provide rural residents with more specialized medical and health information, contributing strong information support for the development of rural medical and health care, and to a certain extent, reducing the economic burden of farmers’ medical and health care (21, 22).

Farmers can obtain efficient medical and health services and products through online consultation, online purchase of medicines, and telemedicine, which prompt rural residents with limited economic conditions and scarce medical resources to enjoy the high-quality medical resources of tertiary hospitals. Guiding the sinking of quality medical resources into rural areas and prompting Internet consultations to “replace” or relieve the workload of primary health-care organizations can continuously improving the efficiency of rural medical services, which avoid the poor decision-making behaviors of farmers who give up medical treatment because of a lack of economic resources. Accordingly, the following two research hypotheses are proposed:

*H1a:* Internet medical information overflow has a “crowding-in effect” on farmers’ medical and healthcare consumption.

*H1b:* Internet medical information overflow has a “crowding-out effect” on farmers’ medical and healthcare consumption.

#### 2.1.2 A study of the mediating role of healthcare behavior in the relationship between internet medical information spillovers and farmers’ healthcare consumption

In recent years, although the Internet has broadened the ways and channels for farmers to obtain medical and health information, there exists the phenomenon of too much information and the quality is worrying, which will affect the patients to make the most appropriate choice of medical behavior, and ultimately increase the farmers’ medical and health consumption expenditure (23). Cho and Sarvary et al. introduced the quality signal game model into the medical service field, arguing that patients have a strong willingness to demand quality signals of medical and health information in order to break the asymmetric information interaction between doctors and patients, and that the Internet medical information has a strong guiding effect on patients’ medical behavior (24, 25). Some scholars believe that the spillover effect of Internet medical information can alleviate the imbalance and asymmetry of medical information, prevent farmers from mistakenly entering the blind spot of knowledge about common diseases, and guide farmers to choose the appropriate medical institutions according to their conditions (26). However, most scholars believe that access to information about healthcare services on the Internet does not mean that healthcare “uncertainty” has been eliminated. Disease severity, potential consequences, and estimated costs still need to be judged and assessed based on offline visits by residents. It is difficult to fully utilize and effectively analyze the medical information exchanged through the Internet by both doctors and patients. This not only places the study of health care costs in the era of information technology development, but also enriches the literature on the social effects of information technology in rural areas, and provides new ideas on how to reduce the health care costs of farmers under the service model of “Internet+Healthcare.” It can be seen that the explanation of excessive medical health consumption by Internet medical information spillover highlights the importance of rational choices in medical behavior even more (27). Therefore, the following research hypotheses is proposed:

*H2:* Medical behavior plays a “mediating role” between Internet medical information spillover and farmers’ healthcare consumption.

#### 2.1.3 A study of the mediating role of economic capital in the relationship between internet medical information spillovers and farmers’ healthcare consumption

The most important and fundamental form of capital, first defined by Bourdieu, is economic capital, and that economic capital is a key factor in limiting the formation of effective decision-making behavior in individuals (28). It has been shown that the effect of economic factors on residents’ healthcare consumption is more prominent than institutional factors (29). Therefore it is more worthwhile to think about what role farmers’ economic capital plays in Internet medical information spillover and farmers’ healthcare consumption. With the integration and development of digital villages and healthy villages, the overflow of Internet medical information has brought great opportunities for the development of rural health. Internet medical information overflow promotes the equalization of urban and rural high-quality medical and health resources, continuously improves farmers’ health literacy, which provides high-quality human capital for rural development, and promotes the rapid upgrading of the rural economy (30), thus unleashing the potential of farmers’ healthcare consumption. It has been found that Internet information has a significant impact on the health of the population, but the economic man hypothesis makes it difficult to align the interests of Internet healthcare service companies and the healthcare interests of the population (31), which reduces the quality of medical information on the Internet and affects the health literacy of the population, ultimately creating the phenomenon of “two lows” in terms of the quantity and quality of labor supply. Conversely, high-quality Internet healthcare resources can improve healthy human capital (26). Human capital accumulation, in turn, leads to increasing agricultural productivity to enhance farmers’ economic capital, which in turn contributes to a reduction in the incidence of rural poverty (32). At the same time, Connected Payments can open up medical credit channels through Internet medical information, which is conducive to financing more healthcare expenditures in a short period of time, thus easing the pressure of consumption after large healthcare expenditures. Therefore, the Internet medical information overflow can directly or indirectly improve the economic structure of farmers’ households, and lay the economic foundation for farmers to cope with health emergencies and major illnesses and treatments (33, 34), which preventing the vicious cycle of “poverty causing illness and returning to poverty” in rural households. Therefore, the following research hypotheses are proposed:

*H3:* Economic capital “mediates” the relationship between Internet medical information spillovers and farmers’ healthcare consumption.

**Figure 1:**
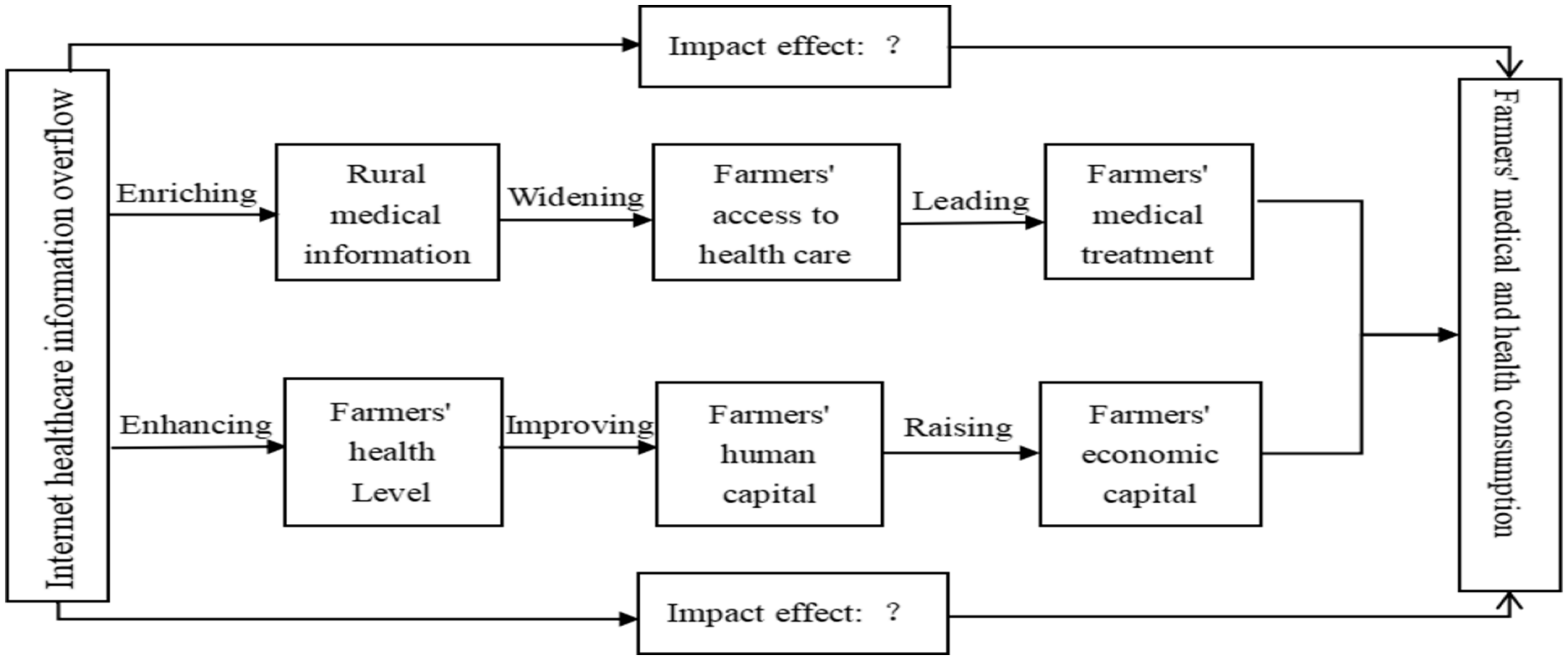
Framework for analyzing the impact mechanism of farmers’ healthcare consumption in the context of Internet healthcare information spillover scenario.

In summary, it is argued in this paper that the uncertainty and commercial nature of Internet healthcare information spillover may induce farmers’ healthcare behavior. In turn, the public good of Internet healthcare information overflow may guide farmers to choose appropriate healthcare behaviors, which in turn affects healthcare consumption. At the same time, the overflow of high-quality Internet medical information can enhance farmers’ health and improve their health human capital, thus strengthening their economic capital. As farmers’ economic income levels rise, healthcare spending power will also be unleashed. Based on this, a framework is constructed for analyzing the influence mechanism of farmers’ healthcare consumption in the context of Internet medical information overflow (Figure 1).

### 2.2 Data sources

The data for this study come from the China Family Tracking Survey (CFPS), a national comprehensive social tracking survey program implemented by the China Social Science Survey Center (ISSS) at Peking University. CFPS collects data from different individuals, families and communities to reflect China’s economic, social, demographic, educational and health changes, providing a solid data base for academic research and policy decisions. The latest CFPS2020 survey data is used in order to analyze the relationship and influence mechanism of Internet medical information spillover on farmers’ healthcare consumption. The healthcare consumption of minors under the age of 18 and the older adult over the age of 65 may not be influenced by the overflow of Internet healthcare information. Because most minors under the age of 18 do not yet have the ability to consume health care independently and fully recognize the validity of Internet medical information. The majority of older people over 65 years of age rely on the household economy for their healthcare consumption, and due to their low digital health literacy, more decisions are made by their guardians or family members. In order to avoid any impact on the generalizability of the study, therefore, farmers aged 18–65 years were selected for the study in this paper (19). Some invalid and missing responses are eliminated based on the characteristics of the study variables and the final valid sample size of 11,647 was obtained after collation.

### 2.3 Selection of variables

#### 2.3.1 Explained variables

Healthcare consumption is the explanatory variable of this paper. Based on the principle of data availability and the purpose of the study, the CFPS original questionnaire on household health care expenditures in the past 12 months was selected, which is the sum of household medical and health care expenditures over the past 12 months. In this paper, on the basis of obtaining the amount of household health care consumption expenditure, it is divided by the number of household size to obtain the amount of per capital health care consumption expenditure of farmers. In order to reduce the research error, the logarithm of the amount of per capital health care consumption expenditure of farmers was taken to represent their health care consumption.

#### 2.3.2 Core explanatory variables

Internet healthcare information spillover is the core explanatory variable of this paper. The CFPS questionnaire was not designed to address access to Internet medical information separately. Therefore, the research ideas of Chen Liu, Hai Gu, and Junhao Wang (19, 26, 35), etc. were drawn on, and “the importance of accessing information on the Internet” reported in the original CFPS questionnaire was chosen as the core explanatory variable, i.e., the proxy variable for information spillover in Internet healthcare. The assignments are: very unimportant = 1, unimportant = 2, average = 3, important = 4, very important = 5.

#### 2.3.3 Mediating variables

Medical attendance behavior and economic capital is mediating variables in this paper. In this paper, the question “Where do you usually go if you go to the doctor?” was chosen to represent the mediator variable “medical care behavior.” This question represents the mediator variable, “medical care behavior.” The assignments are: clinic = 1; community health service station/village health office = 2; community health service center/township health center = 3; specialty hospital = 4; general hospital = 5. Second, the net household income reported in the original CFPS questionnaire was selected and divides it by the number of household size to finally obtain the net household income per capital. To minimize research error, net household income per capital is logarithmic to represent “economic capital.”

#### 2.3.4 Control variables

Based on existing studies and the original CFPS questionnaire, 10 variables that may affect farmers’ healthcare consumption were selected, including age, gender, marital status, years of education, health status, social health insurance, chronic diseases, alcohol consumption, access to healthcare, and level of access to healthcare, from the perspective of individual characteristics, economic factors, and potential demand for healthcare, as control variables. Table 1 reports the variable descriptions and descriptive statistics results.

**Table 1:** Reports the variable descriptions and descriptive statistics results.

Variable category	Variable name	Symbol	Variable definition	Mean	Standard deviation	Minimum	Maximum
Explanatory variables	Healthcare consumption	med	Log healthcare consumption per household	5.4006	2.6173	0	11.9184
Core explanatory variables	Internet healthcare information spillover importance	int	Very unimportant = 1, Unimportant = 2, Average = 3, Important = 4, Very important = 5	3.5726	1.4751	1	5
Mediator variable	Health-care behavior	beh	Clinics = 1; community health service stations/village health units = 2; community health service centers/township health centers = 3; specialized hospitals = 4; general hospitals = 5	2.9317	1.5873	1	5
	Economic capital	finc	Log net household income per capital	9.7075	0.9933	0	14.5142
Control variable	Age	age	Actual value	41.9086	13.3198	18	65
	Genders	gen	Female = 0; Male = 1	0.5006	0 0.5000	0	1
	Educational attainment	edu	Actual value	8.5216	4.4339	0	24
	Marital status	mar	Unmarried, widowed, divorced, cohabiting = 0; married (with spouse) = 1	0.7858	0.4103	0	1
	Health status	heal	Very healthy = 1; Very healthy = 2; Fairly healthy = 3; Average = 4; Unhealthy = 5	2.8309	1.1975	1	5
	Social health insurance	m-ins	With public medical care, urban workers’ basic medical insurance, urban residents’ basic medical insurance or new rural cooperative medical care = 1; No = 0	0.9053	0.2928	0	1
	Have a chronic disease in the past 6 months	chr	Yes = 0; No = 1	0.8761	0.3295	0	1
	Drinking alcohol 3 times a week in the past month	dri	Yes = 0; No = 1	0.8759	0.3297	0	1
	Access to health care	sat	Very dissatisfied = 1; Dissatisfied = 2; Fair = 3; Satisfied = 4; Very satisfied = 5	3.7629	0.7667	1	5
	Level of access to health care	lev	Very bad = 1; Bad = 2; Fair = 3; Good = 4; Very good = 5	3.6162	0.9152	1	5

### 2.4 Methods

#### 2.4.1 Ordinary least Square

To examine the impact of Internet medical information spillover on farmers’ healthcare consumption, Internet medical information spillover was taken as the core explanatory variable and healthcare consumption was taken as the explanatory variable. For the one continuous variable, healthcare consumption, a linear regression of the entire sample can be performed using ordinary least squares (OLS). And there are many factors that affect the explained variable healthcare consumption, so the OLS model (21) is set as follows Equation (1):


(1) $$\mathrm{me}d_{i}=\alpha_{0}+\alpha_{1}{\mathrm{int}}_{i}+\alpha_{2}X_{i}+\varepsilon_{i}$$

where $\mathrm{me}d_{i}$ is the explanatory variable, representing the ith farmer’s healthcare consumption, ${\mathrm{int}}_{i}$ is the explanatory variable Internet healthcare information spillover, $X_{i}$ is the control variable, $\alpha_{0},\;\alpha_{1}\text{,}$ $\alpha_{2}$ are the regression coefficient, and $\varepsilon_{i}$ represents the residual term.

#### 2.4.2 Methods for mediating effect analysis

Medical behavior. This study refers to the intermediary model constructed by Wen Zhonglin et al. (36), and the following Equations (2), (3), (4) are built. First, the impact of Internet healthcare information spillovers on farmers’ healthcare consumption is to be tested. Second, the impact of Internet healthcare information spillover on farmers’ healthcare behavior will be tested. Finally, Internet medical information overflow, medical care behavior, and medical health consumption are to be put into the model at the same time to test the relationship between the influence of Internet medical information overflow, medical care behavior and medical health consumption.


(2) $${med}_{i}=\alpha_{0}+\alpha_{1}{\mathrm{int}}_{i}+\alpha_{2}X_{i}+\varepsilon_{i}$$

(3) $$\mathrm{be}h_{i}=\theta_{0}+\theta_{1}{\mathrm{int}}_{i}+\varepsilon_{i}$$

(4) $$\mathrm{me}d_{i}=\gamma_{0}+\gamma_{1}{\mathrm{int}}_{i}+\gamma_{2}\mathrm{be}h_{i}+\gamma_{3}X_{i}+\varepsilon_{i}$$

Economic capital. In this study, the following Equations (5), (6), (7) are constructed with reference to the mediation model constructed by Wen Zhonglin *et al* (36). First, the impact of Internet healthcare information spillovers on farmers’ healthcare consumption should be tested. Second, the impact of Internet healthcare information spillovers on economic capital will be examined. Finally, Internet medical information spillover, economic capital, and healthcare consumption are put into the model at the same time to test the relationship between the influence of Internet medical information spillover, economic capital, and healthcare consumption.


(5) $$\mathrm{me}d_{i}=\alpha_{0}+\alpha_{1}\mathrm{fin}c_{i}+\alpha_{2}X_{i}+\varepsilon_{i}$$

(6) $${\mathrm{finc}}_{i}=\beta_{0}+\beta_{1}{\mathrm{finc}}_{i}+\varepsilon_{i}$$

(7) $${med}_{i}=\varphi_{0}+\varphi_{1}{\mathrm{finc}}_{i}+\varphi_{2}X_{i}+\varepsilon_{i}$$

## 3 Results

### 3.1 Baseline regression analysis

Before conducting the baseline regression, a multicollinearity test for each variable is performed. The mean value of the variance inflation factor VIF among the variables is 1.34 and the maximum value is 1.85, which are less than 5, indicating that there is no serious problem of multicollinearity among the variables (37). In this paper, the OLS model is used to estimate the parameters of the regression coefficients of the Internet medical information spillover affecting farmers’ medical and healthcare consumption. The parameter estimates of the benchmark regression model are shown in Table 2. Models 1–3 show the regression results with the stepwise addition of individual characteristics, economic factors, and potential medical needs. As can be seen from Table 2, r2 is increasing, indicating that the model fit is gradually optimized.

**Table 2:** Impact of Internet medical information spillover on farmers’ healthcare consumption.

Variables and statistical parameters	Model 1	Model 2	Model 3
int	0.0458**	0.0437**	0.0449**
	(0.0184)	(0.0184)	(0.0183)
age	0.0034	0.0026	0.0005
	(0.0025)	(0.0025)	(0.0025)
gen	−0.0495	−0.0512	−0.0176
	(0.0490)	(0.0490)	(0.0516)
edu	0.0279***	0.0268***	0.0269***
	(0.0066)	(0.0066)	(0.0066)
mar	0.1916***	0.1646**	0.1784***
	(0.0669)	(0.0672)	(0.0673)
heal	0.3325***	0.3322***	0.2744***
	(0.0209)	(0.0209)	(0.0219)
m-ins		0.3336***	0.3213***
		(0.0829)	(0.0827)
chr			−0.6320***
			(0.0780)
dri			0.1576**
			(0.0770)
sat			−0.0200
			(0.0375)
lev			−0.0527*
			(0.0315)
_cons	3.7916***	3.5618***	4.4712***
	(0.1601)	(0.1699)	(0.2485)
r2	0.0253	0.0267	0.0329
N	11,647	11,647	11,647

Note *, **, *** represent 10, 5, and 1% significance levels, respectively; numbers in parentheses represent standard errors (same below).

As can be seen from Table 2, the overflow of Internet medical information significantly and positively affects farmers’ healthcare consumption at the 1% level, indicating that the overflow of Internet medical information increases farmers’ healthcare consumption and produces a “crowding-in effect,” accepting hypothesis H1a and rejecting hypothesis H1b. The reason why the overflow of Internet medical information increases farmers’ healthcare consumption may be due to farmers’ low health literacy, which makes it difficult for them to recognize the value of Internet medical information, and the difficulty of determining the severity of a medical condition by means of online communication, which leads to patients’ physical health anxiety and then over-consumption of medical and healthcare services. In terms of individual characteristics, education level, marital status, and health status all significantly and positively affect healthcare consumption. The more educated farmers pay more for healthcare consumption, probably because they are more concerned about their health and have more financial means to pay for healthcare services. Married farmers may be subjected to their spouses’ care and supervision of their health for longer periods of time and incur more healthcare consumption. Neither age nor gender significantly affects healthcare consumption, possibly because the sample selected for this paper was aged 18–65 years old. In rural areas, where the modern idea of “equality between men and women” is gradually replacing the traditional idea of “preference for men over women,” there are no significant gender differences in medical and health care consumption. In terms of economic factors, social health insurance positively and significantly affects farmers’ health-care consumption, possibly because farmers with health insurance have lower out-of-pocket health-care expenditures, which releases their health-care consumption demand. In terms of potential healthcare demand factors, such as the presence of a chronic disease in the past 6 months and having drunk alcohol three times a week in the past month, the level of access to healthcare significantly influences healthcare consumption, suggesting that farmers who drink alcohol perceive themselves as being in better health than those who do not drink alcohol (37). So farmers who drink at least three times a week do not pay high healthcare spending. Farmers without chronic diseases spend less on health care than those with chronic diseases. The higher the level of point-of-care access, the more farmers spend on health care consumption. Whereas, the condition of access to health care does not have a significant impact on farmers’ health care consumption, probably because farmers are more concerned with curing patients’ diseases than with the comfort of the point of access to health care.

### 3.2 Robustness tests

In order to ensure the reliability and accuracy of the research findings, it is necessary to conduct a robustness test of the benchmark regression model in this paper. And there are various methods of robustness testing, while this paper chooses to replace the core explanatory variables and adjust the sample period for robustness testing. First, replace the core explanatory variables. In this paper, the two variables “whether to access the Internet on mobile” and “whether to access the Internet on computer” in the CFPS questionnaire were selected and merged into “whether to access the Internet” (inter) as a substitute for the core explanatory variable. Inter takes the value of 1 when the farmer chooses mobile internet access or computer internet access, otherwise it takes the value of 0. Second, the sample period was adjusted. Since the impact of Internet medical information spillover on farmers’ healthcare consumption is lagged, one period of lagged data (CFPS2018) was adopted to conduct robustness tests. As can be seen in Table 3, the core explanatory variables have a positive and significant impact on farmers’ healthcare consumption, both by substituting variables and adjusting for sample period.

**Table 3:** Robustness tests.

Variables and statistical parameters	Replacement of core explanatory variables	Adjustment of sample period
int	–	0.0639^***^ (0.0139)
inter	0.2667^***^ (0.0749)	--
Control variable	Yes	Yes
_cons	4.6199^***^ (0.2357)	4.6043^***^ (0.1839)
r2	0.0335	0.0365
N	11,647	16,156

### 3.3 Endogeneity test

In empirical analysis, omitted variable errors are unavoidable, and omitted variable errors can lead to endogeneity problems, which affect the estimates between the core explanatory variables and the explained variables. In order to solve the endogeneity problem that may exist in the article, the ideas of Jingli et al. (38) and Yang et al. (39) were drawn on. Based on the description of the CFPS questionnaire, “telecommunication communication cost” (iv) was selected as the instrumental variable in this paper. Telecommunication communication costs are incurred by rural residents who choose the appropriate network payment method according to their family’s financial situation and level of need. The reason for choosing “telecommunication cost” as an instrumental variable is that whether Internet medical information is accepted or utilized by rural residents has a certain correlation effect with communication consumption behavior, but it does not determine farmers’ medical and health care consumption, which satisfies the condition of exogeneity of the instrumental variable. Table 4 reports the results of the two-stage least squares regression using instrumental variables. The regression results show that the Kleibergen-Paap rk LM statistic is significant at the 1% level, which suggests a rejection of the original hypothesis that instrumental variables are not identifiable. Both the Cragg-Donald Wald F statistic and the Kleibergen-Paap rk Wald F statistic are much larger than the critical value of 16.38 at the 10% level, rejecting the original hypothesis that the instrumental variables are weakly identified. Therefore, from the regression results, the instrumental variables selected in this paper have strong rationality. Meanwhile, the positive effect of instrumental variables on farmers’ healthcare consumption is unchanged, which is largely consistent with the previous findings. The results of the two-stage least squares regression further confirm the robustness of the conclusion that Internet medical information spillovers have a “crowding-in effect” on farmers’ healthcare consumption.

**Table 4:** Instrumental variable regression results: 2SLS.

Variables	Phase I	Phase II
int	–	2.0164^***^ (0.3388)
iv	0.1092^***^ (0.0030)	–
Control variable	Yes	Yes
_cons	3.7455^***^ (0.1445)	−4.096^***^ (1.5130)
r2	0.2247	0.0063
N	11,647	11,647
Kleibergen-Paap rk LM statistic	65.765^***^	
Cragg-Donald Wald F statistic	40.4467 [16.38]	
Kleibergen-Paap rk Wald F statistic	66.5201 [16.38]	

The null hypothesis of the KleibergenPaap rk LM test is that the instrumental variables are under-identified, if the null hypothesis is rejected then the instrumental.

variables are justified. The null hypothesis of the Cragg-Donald Wald F and Kleibergen-Paap rk Wald F tests is that the instrumental variables are weakly identified, and if the null hypothesis is rejected the instrumental variables are justified. Values in brackets [] in the Cragg-Donald Wald F and Kleibergen-Paap rk Wald F tests are critical values for the Stock-Yogo test.

### 3.4 Heterogeneity analysis

According to the Statistical Report on the Development Status of the Internet in China, Internet information acquisition has long presented a youthful trend. To highlight the existence of differences in the impact of different age groups, the impact of Internet medical information spillover on farmers’ healthcare consumption is analyzed from the perspective of age. In accordance with the new age segmentation proposed by the United Nations World Health Organization (WHO), the age of the study sample was divided into three levels: the young group (18–44 years), the middle-aged group (45–59 years), and the young-older group (60–65 years). As can be seen from Table 5, the percentage of farmers aged 18–44 years accessing medical information on the Internet is 53.4387%, the percentage of farmers aged 45–59 years is 36.8593%, and the percentage of farmers aged 60–65 years is 9.7021%. Internet healthcare information spillover did not significantly affect farmers’ healthcare consumption in the youth group. However, it positively and significantly affects the healthcare consumption of farmers in the middle-aged and young middle-aged and old-aged groups, which may be due to the fact that, compared to farmers in the middle-aged and young middle-aged and old-aged groups, the rural youth group has a better knowledge structural system and information discernment ability, which does not result in excessive induced healthcare consumption due to the overflow of Internet healthcare information. The rural middle-aged and older adult groups’ ability to accept new things is not as fast as that of the youth groups, and they are unable to effectively identify the practical value of Internet medical information, which makes it easy to produce induced medical and health care consumption.

**Table 5:** Impact of Internet medical information spillover on farmers’ healthcare consumption: age grouping result.

Variables and statistical parameters	Youth group	Middle-aged group	Young-old group
int	0.0154 (0.0206)	0.0692^***^ (0.0255)	0.1199^**^ (0.0497)
Control variable	Yes	Yes	Yes
_cons	4.7375^***^ (0.3858)	2.0727^***^ (0.0.6365)	4.5777 (2.966)
r2	0.0256	0.0518	0.0526
N	6,224	4,293	1,130

### 3.5 Exploration of impact mechanisms

#### 3.5.1 Access to health care

Considering that rural Internet medical information spillover may affect farmers’ healthcare consumption by guiding the choice of medical care, medical care behavior (beh) is selected as a mediator variable to carry out an empirical analysis of the mediation effect, and the regression results are shown in Table 6. In column (2) of Table 6, the regression result of rural Internet medical information spillover is significantly positive, indicating that Internet medical information spillover has a significant positive impact on farmers’ health care behavior, probably because complete, asymmetric, and commercialized Internet medical information exacerbates the ambiguity of farmers’ knowledge of diseases and causes rural patients to have panic feelings about their health. Moreover, most of China’s high-quality medical and healthcare resources are not fully tilted to rural areas, exacerbating the inducing effect of Internet medical information on farmers’ medical behaviors, thus leading to the “siphoning phenomenon” of Chinese farmers’ medical behaviors (9). Column (3) of Table 6 contains regression results for both Internet healthcare information spillover, healthcare-seeking behavior, and healthcare consumption. Compared with column (1) of Table 6, the regression coefficient of rural Internet medical information overflow has decreased and is still significantly positive, which indicates that although the Internet medical information overflow has improved farmers’ basic knowledge of diseases, the complexity and uncertainty of the Internet medical information has increased the risk of misdiagnosis of common diseases, which induces farmers to choose medical treatment by showing the phenomenon of “tendency to be high” in order to increase healthcare consumption. In summary, the medical care behavior plays a mediating role between Internet medical information spillover and farmers’ healthcare consumption, and hypothesis H2 is verified.

**Table 6:** Intermediary mechanism test: medical attendance behavior.

Variables and statistical parameters	(1)	(2)	(3)
	Med	Beh	Med
int	0.0449**	0.0258^***^	0.0445^**^
	(0.0183)	(0.0100)	(0.0183)
beh	--	--	0.0759^***^
			(0.0153)
age	0.0005	--	0.0005
	(0.0025)		(0.0025)
gen	−0.0176	--	−0.0173
	(0.0516)		(0.0516)
edu	0.0269***	--	0.0237^***^
	(0.0066)		(0.0066)
mar	0.1784***	--	0.1823^***^
	(0.0673)		(0.0672)
heal	0.2744***	--	0.2679^***^
	(0.0219)		(0.0219)
m-ins	0.3213***	--	0.3147^***^
	(0.0827)		(0.0826)
chr	−0.6320***	--	−0.5898^***^
	(0.0780)		(0.0784)
dri	0.1576**	--	0.1649^**^
	(0.0770)		(0.0769)
sat	−0.0200	--	−0.0207
	(0.0375)		(0.0375)
lev	−0.0527*	--	−0.0660^**^
	(0.0315)		(0.0316)
_cons	4.4712***	2.8396^***^	4.3056^***^
	(0.2485)	(0.0385)	(0.2505)
r2	0.0329	0.0006	0.0350
N	11,647	11,647	11,647

#### 3.5.2 Economic capital

Considering that rural Internet medical information spillover may affect farmers’ medical and health consumption by enhancing farmers’ economic capital, economic capital (finc) is selected as a mediating variable in order to carry out empirical analysis of mediation effect. Considering the limitations of the CFPS survey data, the logarithm of net household income per capital is used as a proxy variable for economic capital, and the regression results are shown in Table 7. In column (2) of Table 7, the regression result for rural Internet healthcare information spillover is significantly positive, indicating that Internet healthcare information spillover has a significant positive impact on farmers’ economic capital. It can be seen that the overflow of Internet medical information will increase the accumulation of farmers’ economic capital, thus releasing farmers’ demand for medical and health care consumption and helping to alleviate the economic dilemma of “no treatment for minor illnesses and no treatment for major illnesses.” Column (3) of Table 7 contains regression results for both Internet healthcare information spillovers, economic capital, and healthcare consumption. Compared with column (1) of Table 7, the regression coefficient of rural Internet medical information spillover decreases and remains significantly positive, which indicates that rural Internet medical information spillover under the technological dividend is conducive to the improvement of rural medical and health services to reduce the redundant costs of farmers’ out-of-town medical care and information searching, and also conducive to the enhancement of farmers’ health literacy to improve farmers’ human capital and accumulate more economic capital. While Internet medical information spillover adds complexity and challenge to regulating farmers’ healthcare consumption, it also relaxes the economic parameters of farmers’ healthcare choices (22), to meet the diverse healthcare consumption needs of farmers. In summary, economic capital plays a mediating role between Internet healthcare information spillovers and farmers’ healthcare consumption, and hypothesis H3 is tested.

**Table 7:** Inter-mediation mechanism test: economic capital.

Variables and statistical parameters	(1)	(2)	(3)
	Med	Finc	Med
int	0.0449**	0.1201^***^	0.0363^**^
	(0.0183)	(0.0061)	(0.0184)
finc	--	--	0.1283^***^
			(0.0250)
age	0.0005	--	0.0004
	(0.0025)		(0.0025)
gen	−0.0176	--	−0.0192
	(0.0516)		(0.0516)
edu	0.0269***	--	0.0201^***^
	(0.0066)		(0.0067)
mar	0.1784***	--	0.1741^***^
	(0.0673)		(0.0672)
heal	0.2744***	--	0.2754^***^
	(0.0219)		(0.0219)
mins	0.3213***	--	0.3207^***^
	(0.0827)		(0.0826)
chr	−0.6320***	--	−0.6314^***^
	(0.0780)		(0.0779)
dri	0.1576**	--	0.1690^**^
	(0.0770)		(0.0769)
sat	−0.0200	--	−0.0177
	(0.0375)		(0.0375)
lev	−0.0527*	--	−0.0538^*^
	(0.0315)		(0.0315)
_cons	4.4712***	9.2785^***^	3.3071^***^
	(0.2485)	(0.0237)	(0.3362)
r2	0.0329	0.0318	0.0351
N	11,647	11,647	11,647

## 4 Discussion

Using data from the China Household Tracking Survey, this paper analyzes the impact of Internet medical information spillover on farmers’ medical and healthcare consumption through benchmark regression. Considering the issues of robustness and endogeneity, the core explanatory variables were replaced by “whether or not to access the Internet” and the sample period was adjusted to re-run the regression analysis, and telecommunication costs were selected as an instrumental variable to test the robustness and reliability of the findings. Considering the age group heterogeneity, the study sample was divided into young, middle-aged, and young-aged groups for heterogeneity analysis. Finally, the mechanism of action between Internet medical information spillover and farmers’ healthcare consumption is tested by constructing a double mediation effect model. The results of this pioneering study will provide reference suggestions for public health policy makers to alleviate the pressure on farmers’ medical and healthcare consumption, enhance the service level of primary healthcare providers, and improve the hierarchical diagnosis and treatment system in the context of the Internet.

First, Internet healthcare information spillover has a significant positive impact on healthcare consumption in the rural middle-aged and young-aged groups, while it is insensitive to the impact on healthcare consumption in the young-aged group. This indicates that the quality of medical information on the Internet varies and is prone to cause asymmetry of information between doctors and patients, which leads to doctors exaggerating the conditions of illnesses and inducing farmers, especially middle-aged and older adult farmers, to spend too much on medical expenses. National policies should focus on accelerating the sinking of high-quality Internet medical resources to rationally guide farmers’ medical and healthcare consumption. On one hand, the government should improve the implementation mechanism of village-level Internet medical policies. Due to historical reasons and economic conditions, it is difficult to implement China’s Internet healthcare policy in rural areas, but the effective implementation of the policy is a key measure to promote the full equalization of Internet healthcare at the village level. Therefore, China should face up to the urban–rural gap, grasp the actual situation in rural areas, promote the modernization of the capacity of the main body of policy implementation, improve the multifaceted means of policy implementation based on Internet technology, and enhance the sense of acceptance of the clients of policy implementation. On the other hand, the quality of “Internet+Healthcare” services should be improved in primary healthcare organizations. The lack of “good specialists” as well as hardware and software resources in rural areas are the main reasons why farmers question the conditions of access to health care in the townships. Therefore, it is necessary to attract excellent medical service talents to go deep into the grassroots and drive the flow of digital technology to the rural areas, so as to avoid the decline in the efficiency of medical and health services in the rural areas as a result of the digital divide between urban and rural areas, and, at the same time, to reduce the redundancy costs incurred by going out to seek medical treatment for serious illnesses. What’s more, rural middle-aged and older adult residents should scientifically draw medical information from the Internet. With the two-way impact of population aging and rural hollowing out, the focus and difficulty of rural Internet medical service is to improve the Internet information access and discrimination ability of middle-aged and old-aged groups. Therefore, it is necessary to carry out basic Internet technology training for middle-aged and old-aged groups, push vivid and interesting medical information and easy-to-understand short medical videos through popular Internet platforms such as WeChat, Jitterbug and Shutterbug, which make them realize the convenience and enticement of Internet medical information, improve their degree of use of medical information and their ability to identify medical information, and realize the integration of grass-roots consultation and Internet consultation, so as to reduce the cost of consultation and overmedication among the middle-aged and old-aged people in the countryside.

Second, Internet healthcare information spillovers act on farmers’ healthcare consumption through healthcare behaviors. Specifically, Internet healthcare information overflow may cause doctors to interfere with farmers’ healthcare behavior, which may lead to more healthcare consumption by farmers. Therefore, national policies should focus on optimizing the “Internet + Healthcare” service to guide farmers to make reasonable choices about medical care. First, the government should optimize the “Internet + county medical community.” The focus and difficulty of hierarchical care lies in the choice of care for county and township residents. The lead hospital should monitor the operation and management of the organizations within the medical community in real time, and carry out activities such as remote consultation, remote surgical guidance, academic discussions, expert lectures and training of grassroots talents to the grassroots level on a regular or emergency basis, so as to improve the quality of medical and healthcare services within the county area. Primary institutions should upload patients’ health records and electronic medical records, etc. to the lead hospital in a timely manner according to statistical standards, and collaborate to establish an internal information linkage mechanism within the medical community to promote the integration and sharing of medical resources within the medical community. Secondly, contracted family doctors should give full play to their functions in order to facilitate hierarchical diagnosis and treatment. The “gatekeeper system” should guide rural residents to seek medical treatment in accordance with the degree of illness, so as to prevent primary health-care institutions from being reduced to “pharmacies.” Family doctors regularly check the utilization of Internet medical information by rural residents, so as to prevent Internet medical information from inducing farmers, especially the middle-aged and older adult, to over-consume, and to accelerate the formation of a hierarchical treatment pattern in which “minor illnesses are treated at the grass-roots level, major illnesses are treated in hospitals, and rehabilitation is carried out back at the grass-roots level.” Third, primary healthcare organizations should make full use of the “Internet + medical service” platform to improve their service quality. Primary medical institutions can make full use of the Internet platform to improve telemedicine services and Internet medical consultation, breaking the spatial and geographical limitations that make it difficult for specialists to go down to the grassroots, and can carry out Internet education and training for primary medical doctors through the Internet platform, thus improving the capacity of primary medical and healthcare services in the short or long term. In turn, it effectively guides farmers to seek reasonable medical treatment “at the grass-roots level to realize the diagnosis and treatment of most diseases,” and enables farmers to truly enjoy the dividends of science and technology “without going out to ask for diagnosis and treatment of minor ailments.” Fourth, multiple subjects should clarify their own responsibilities to improve the Internet medical regulatory mechanism. The government should introduce relevant laws and policies to popularize scientific medical and health knowledge, which avoid pseudo-scientific health knowledge to mislead the public, and promote medical and health resources tend to “public welfare”; Medical institutions should clarify the main responsibility within the construction of the traceability mechanism, the formation of the whole traceability system, and at the same time utilize online platforms to strictly scrutinize medical and health information closely related to people’s health. Social media should play the role of public opinion to prevent malpractices such as malicious fraud and inducement by medical-related organizations, and enhance the transparency and certainty of Internet medical information.

Third, the spillover of Internet medical information affects farmers’ medical and health consumption through economic capital. Specifically, the spillover of quality Internet healthcare information can enhance farmers’ health literacy to increase their human capital, which in turn accumulates economic capital to unleash demand for healthcare consumption. Therefore, national policies should focus on the importance of the power of the Internet in improving the economic capital of farmers’ access to health care, thereby easing the burden of health care on the population. The first one, grassroots government personnel and farmers should focus on their own digital health literacy, and the government should encourage the development of social medical institutions in rural areas. Primary healthcare organizations should comprehensively improve their online and offline service capabilities to enhance the number of free online health consultations, and open up channels for farmers to access low-cost and effective medical and health information. At the same time, financial incentives can be used by the government to attract a group of medical institutions with Internet technology to rural areas. The “catfish effect” of social capital can lead to breakthroughs in rural medical Internet services, which is not only conducive to enhancing the health capital of farmers, but also conducive to increasing the economic capital of rural medical care, thereby alleviating the livelihood problem of “difficult and expensive access to medical care.” Besides, the government can guide farmers to rationally use the new consumption mode of rural medical and health care. Most farmers are skeptical or even resistant to the changed digital payment methods. However, in reality, the convenience and accessibility of Internet consumer finance can alleviate the consumer pressure after a large healthcare expenditure. Mobile connectivity slows credit constraints by expanding household social networks. Currently, software such as We-Chat and Ali-pay can provide micro-lending and various health insurance services to improve healthcare asset liquidity. When faced with the impact of a major illness, farmers who prefer to save can hardly pay large sums of money for healthcare immediately, so healthcare organizations can provide a flexible consumption model combining health insurance, mobile payment and out-of-pocket expenses according to the financial ability of the patient in order to reduce the out-of-pocket payment ratio of farmers’ healthcare, which is conducive to the farmers’ ability to finance more healthcare expenses in a short period of time. Finally, township governments should give full play to their functions in order to optimize the allocation of health resources. The township government can convene a meeting of villagers’ representatives to explain in depth the content of policies related to “Internet+Medical Health” and distribute relevant publicity materials, so as to make them clear about the “real money” contained in the policies, and thus enhance the medical soft economic capital of the farmers. At the same time, the township government guides farmers to use the Internet and other new technologies to form a rural industrial sales chain, which can increase employment opportunities for farmers, enhance farmers’ economic income, improve the economic situation of farmers’ families, so as to break the economic dilemma of “no treatment for minor illnesses and waiting for death for major illnesses” at the root.

## Data availability statement

The original contributions presented in the study are included in the article/supplementary material, further inquiries can be directed to the corresponding author.

## Author contributions

YM: Conceptualization, Data curation, Formal analysis, Resources, Software, Writing – original draft, Writing – review & editing. CX: Conceptualization, Supervision, Writing – review & editing.
